# An Evidence-Based Review of Medicinal Plants Used for the Treatment of Vaginitis by *Avicenna* in "*the Canon of Medicine*"

**DOI:** 10.31661/gmj.v8i0.1270

**Published:** 2019-05-08

**Authors:** Somayyeh Khalilzadeh, Tahereh Eftekhar, Roja Rahimi, Mozhgan Mehriardestani, Malihe Tabarrai

**Affiliations:** ^1^Department of Persian medicine, School of Persian Medicine, Tehran University of Medical Sciences, Tehran, Iran; ^2^Department of Obstetrics and Gynecology, Tehran University of Medical Sciences, Tehran, Iran; ^3^Department of Traditional Pharmacy, School of Traditional Pharmacy, Tehran University of Medical Sciences, Tehran, Iran

**Keywords:** Vaginitis, Avicenna, Anti-Inflammatory, Antibacterial, Persian Medicine

## Abstract

Vaginitis is one of the most common gynecological problems in reproductive age. Because of the limitations of the conventional drugs, identification of new pharmacological interventions for this disease seems to be necessary. The purpose of this article is to review the medicinal herbs mentioned for the treatment of vaginitis by the great Iranian scientist, Avicenna, in his book "*the Canon of Medicine* " to scientifically demonstrate their effects and their potential to be used as complementary therapies. The medicinal plants listed for vaginitis treatment in "*the Canon of Medicine* " were extracted. The scientific name and English common name of the given medicinal plants were searched in databases including PubMed, Scopus, and Cochrane Library until December 2017 to obtain any in vitro, animal, and clinical evidence related to vaginitis. Various pharmacological activities, including anti-inflammatory, wound healing, antimicrobial, antifungal, analgesic, and anti-prostaglandin E2, have been demonstrated for medicinal plants emphasized by *Avicenna* for vaginitis. Randomized controlled trials (RCTs) on Myrtus reported an improvement in the treatment of bacterial vaginosis. Four RCTs on pomegranate indicated a reduction in inflammatory factors in the patients. Medicinal herbs offered in Herbal Medicine are valuable sources for the treatment of various diseases. Effects and pharmacodynamics having been proved by conventional medicine confirm the effectiveness of these herbs. Therefore, these plants can be used in the treatment of vaginitis thanks to further clinical studies.

## Introduction

As one of the most common clinical problems, vaginitis causes the referral of 28% of women to gynecology clinic [1]. Vaginitis is associated with a high risk of complications, such as preterm labor, urinary tract infections, and pelvic inflammatory disease, as well as infections affecting the uterus and tubes in the embryo transfer and causing infertility. According to the estimates, vaginitis affects 13 million women every year in the United States [2, 3]. Bacterial vaginosis and vulvovaginal candidiasis are the most common forms of vaginal infection in women, and azoles are usually used to treat them; however, only fluconazole has been approved by the Food and Drug Administration (FDA). These treatments fail to reduce the relapse rate of the disease, and their use is difficult due to the systemic and local side effects of azoles and contraindications in the first trimester of pregnancy [4, 5]. Due to these complications and the microbial resistance that is caused daily to these drugs, it is reasonable to propose available and effective drugs. One of the options is the use of traditional drugs that have been popular among people for many years. The great Iranian scientist, *Avicenna* (AD 980–1037), has named vaginitis as “*Sayalan-e-Rahem*” in his book “*the Canon of Medicine*.” The purpose of this article is to review the names of medicinal plants listed in this book to scientifically prove their effects and their potential to be used as complementary therapies [6, 8].

## Search Strategies

The names of medicinal plants listed in the book “*the Canon of Medicine*” for vaginitis were extracted (Table-1). The PubMed, Scopus, and Cochrane Library databases were searched until December 2017. Search terms include “vaginitis,” “vaginosis,” “vaginal discharge,” “antibacterial,” “antifungal,” “inflammation” or “antioxidant” and the name of each plant in the abstract and title. The obtained articles were evaluated for the in vitro, animal, and clinical evidence related to vaginitis. The articles that had an association with vaginitis were included. Only English articles were considered, and duplicate articles were deleted. The articles that were not available as full text, letter to the editor, case reports, and articles that studied several plants were excluded due to interference.

## Results

**figure1:**
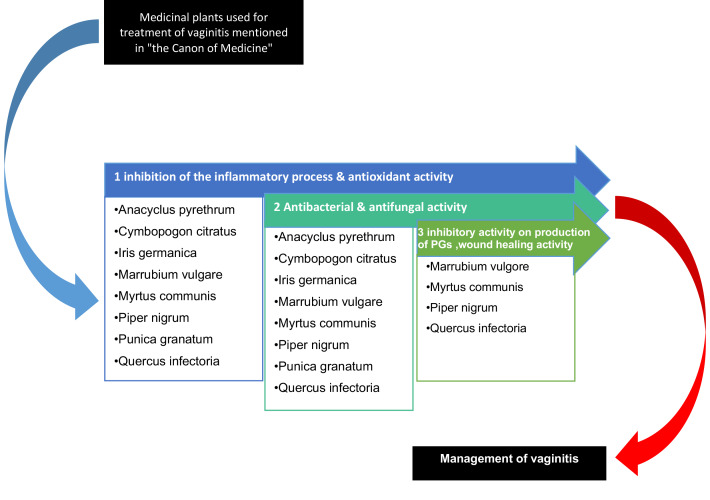
Mechanisms involved in the management of vaginitis by medicinal plants

In Traditional Persian Medicine, the term “*Sayalan-e-Rahem*” refers to any pathological vaginal discharge. Today, vaginitis is one of its most obvious examples. The scientific evidence for the efficacy of the plants listed in “*the Canon of Medicine*” for the treatment of vaginitis including *Anacyclus pyrethrum, Cymbopogon* species,* Iris germanica, Marrubium vulgare, Myrtus communis, Piper species, Punica granatum,* and *Quercus infectoria* has been presented in the articles summarized in Tables 2, 3, and 4. Also, we tried to show some possible mechanisms of them to treatment vaginitis (Figure-1).

### A. pyrethrum (L.) Link

Regarding the in-vitro study of A. pyrethrum (L.) Link, 50% methanol extract of this plant exhibits free radical scavenging; thus, protecting the DNA damage [9]. Moreover, essential oil of its aerial parts is active against *Candida albicans* and *Staphylococcus aureus*[10]. The aqueous and alcoholic extracts of this plant significantly reduce the induced inflammation in mice and rats [11]. A. pyrethrum (L.) Link root and apple extracts used orally in mice have anti-inflammatory properties and reduce the sensation of pain [12].

### ‎Cymbopogon. species

In experimental models, lemongrass polysaccharides regulate the immune system and have anti-tumoral effects [13]. Also, the crude extracts of the plant have antimicrobial activity against *Propionibacterium acnes* and *S. epidermidis* (causes of acne) [14]. The volatile oil of this plant produces anti-inflammatory and antifungal activity [15], and its antioxidant properties have been proven by in vitro and animal models [16, 17]. Injection of *C. schoenanthus* essential oil into the mice after casein-induced inflammation in peritoneum suppresses the accumulation of leukocytes and reduces inflammation [18]. The active parts of this plant in the animal model reduce pain, fever, and inflammatory activity [19, 21]. The methanolic extract of lemongrass in the laboratory model on peripheral blood mononuclear cells can strongly inhibit interleukin-1β [22].

### I. germanica L.

The Iris root is an isoflavonoid-rich source with antimicrobial and anti-mutagenic properties. Also, its compounds can inhibit the activity of alpha-amylase, which can inhibit glucose uptake or accelerate the production of glycogen in the liver, thereby reducing the blood sugar levels in diabetics [23, 25]. Anti-inflammatory, antimalarial, and anticancer effects of *I. germanica* have also been proven. *I. germanica* fails to inhibit *C. albicans*, but it is effective in the treatment of bacterial and viral infections [26, 28].

### M. vulgare L.

The various antioxidant effects of *M. vulgare* (white horehound) have been proven in many cell and animal models [29, 32]. A study on the effects of methanolic extract of this plant on human skin fibroblasts indicated the improvement of cell proliferation as one of the phases of wound healing [32]. In the animal model, inter-peritoneal injection of *M. vulgare* ethanolic extract reduces edema and inflammation [33]. In a laboratory study, white horehound has shown strong antibacterial, antifungal, and anti-tumoral effects [34].

### M. communis L.

Active derivatives of *M. communis*L. including myricetin-3-o-galactoside and myricetin-3-o-rhamnoside have anti-genotoxic properties and are involved in apoptosis [35]. Also, in the various laboratory or human models, it has inhibitory effects on prostaglandin E2, in particular, and anti-inflammatory, cytoprotective, and anti-uterine bleeding effects, in general [36, 38]. In the animal model, the aqueous and alcoholic extracts of *M. communis* have anti-nociceptive and anti-inflammatory effects [39]. *M. communis*L. has similar effects to Sulfasalazine in the treatment of the acetic acid-induced inflammatory bowel disease in rats. Also, its volatile oil in immunosuppressive mice has antifungal effects against *C. albicans* [40, 41]. In random clinical trials, comparing metronidazole vaginal gel with its myrtle-based counterpart showed that the latter improved the treatment of bacterial vaginosis [42]. In the case of human recurrent aphthous stomatitis (RAS), the use of the myrtle has been effective [43].

### Piper. species

Piperine is one of the black pepper phytochemicals with anti-inflammatory activity. In the cell and animal models, it inhibits ATP-induced pyroptosis and is expected to be used for the treatment of bacterial infections in the future [44]. Moreover, Piperine can control the inflammatory factors involved in the development of human osteoarthritis [45] and expresses anti-inflammatory and analgesic properties in rats [46]. The black pepper ethanolic extract inhibits mast cell activation in the animal samples and reduces the allergic inflammation as well [47]. It also has anti-metastatic, anti-depressant, hepatoprotective, immune-regulating, anti-thyroid, anti-tumoral, antinociceptive, and anti-inflammatory properties [48, 51]. Ethanol extract of P. guineense seed has a potent antifungal effect in the experimental model, and its oral intake has not been toxic to mice [52].

### P. granatum L.

Pomegranate is an antifungal and antibacterial herb [53, 54]. Administration of the *P. granatum* mouthwash in comparison with placebo reduces total protein content in the mouth and decreases the activity of aspartate aminotransferase that is involved in gingivitis prevention [55]. However, the use of pomegranate juice compared with placebo has anti-inflammatory effects on athletes and reduces their muscle pain [56]. In obese subjects, the pomegranate extract reduces the inflammatory and oxidative stress [57]. One-year use of the pomegranate extract in hemodialysis patients reduces the risk of inflammation [58]. One study on pomegranate extract mouth rinsing effects on 55 cases of gingival bleeding showed its anti-bleeding activity [59].

### Q. infectoria Olive.

In Persian medicine, the extract of *Q. infectoria* gall has been claimed to eliminate excessive vaginal discharge. *Q. brantii* has antibacterial activity in the agar disc diffusion against *S. aureus* species which are resistant to methicillin and cefixime [60]. The hydro-alcoholic extract of oak in the human laryngeal epidermoid carcinoma (Hep-2) cells has strong anti-proliferative effects [61] and reduces the production of interleukins (IL)-6 and IL-8 in the macrophage [62]. Researchers suggest that occupational exposure to wood dust of the oak tree causes inflammatory responses in the body [63]. However, animal studies have not proven the toxicity of *Q. brantii* but its immunomodulatory effects [64, 65]. *Q. infectoria* gall has anti-inflammatory and antioxidant effects on the colitis in mice and anti-dermatophytes in an animal model [66-68]. In the topical treatment of diabetic ulcers, the cream made from the oak extract reduces the diameter of the wound more than the Silver sulfadiazine cream; however, it is not statistically significant [69].

## Discussion

Due to its complications such as preterm labor, urinary tract infections, and pelvic inflammatory disease, vaginitis highly affects women’s life [2]. Some plants mentioned in this article, such as pomegranate and black pepper, are present in people’s food basket. Various functions of these plants, e.g., anti-inflammatory, wound healing, anti-proliferative, antimicrobial, antifungal, and analgesic, can be used to treat vaginitis and reduce symptoms (Tables-2, 3, and 4). *Vaginitis* is caused by a bacterium or a fungus that can be suppressed by most of these plants [14, 24, 42, 44, 54]. The main mechanism for causing vaginitis symptoms such as itching, irritation, and stimulation is inflammation by microorganisms. All of the proposed drugs including *Q. infectoria* Olive, *P. nigrum* L., *A. pyrethrum*L., and *M. vulgare*L. have anti-inflammatory effects in in-vitro and/or *in-vivo* studies [12, 33, 48, 68]. The prostaglandins also play a role in the development of vaginitis. According to studies, some plants or their phytochemicals showed the effects of PGE2 inhibition [37, 45, 54]. The presence of analgesic agents is required to reduce the clinical symptoms of vaginitis, which was proved in several plants mentioned earlier [12, 39, 43, 46]. The most active ingredients of the proposed herbs are flavonoids, alkaloids, and phenols, among which flavonoids are present at the root of the A. pyrethrum [9], the leaves of the *M. vulgare*[31], and the aerial parts of *M. communis*[40]. Pomegranate, myrtle, and oak are plants investigated in many clinical studies, and their efficacy was proved as well. In different RCTs performed on M. communis, there is an improvement in the treatment of bacterial vaginosis and remission of stomatitis, as well as the reduction of menstrual bleeding [38, 42, 43]. In four RCTs, there is a reduction in inflammatory factors due to the pomegranate use [55-58]. In 40 diabetic patients with Wagner grade-1 or -2 ulcers, the use of topical Q. rubra cream caused a further reduction in ulcer diameter compared with placebo [69].

## Conclusion

Medicinal herbs offered in herbal medicine around the world are valuable sources for use in the treatment of various diseases. The great Iranian scientist, Avicenna, in the 11th century mentioned plants for the treatment of vaginitis in his book “*the Canon of Medicine*.” Accordingly, the present article dealt with the effects and pharmacodynamics of these plants. Based on this study, the usefulness of the plants mentioned for the treatment of vaginitis in “*the Canon of Medicine*” was confirmed. Therefore, in the future, these plants can be used in the treatment of diseases such as vaginitis with more clinical studies.

## Acknowledgment

This study has been partially supported by Tehran University of Medical Sciences (grant No. 96-04-86-37034).

## Conflict of Interest

Authors declare no conflict of interest.

**Table1:** Medicinal Plants Used for Treatment of Vaginitis Mentioned in “*the Canon of Medicine*”

**Scientific name(s)**	**Family**	**Common name(s)**	**Name(s) in** ***“Canon of Medicine”*** ** book**
*Anacyclus pyrethrum* (L.) Link	Asteraceae	Pellitory, Spanish chamomile	*Aagirgarha*
*Cymbopogon* *schoenanthus (*L.) Spreng.	Poaceae	Lemongrass	*Izkhir*
*Iris germanica* L.	Iridaceae	Iris, flag	*Irsa*
*Marrubium vulgare* L.	Lamiaceae	Common horehound or White horehound	*Faraasiun*
*Myrtus communis* L.	Myrtaceae	Myrtle	*Aas*
*Piper nigrum* L.	Piperaceae	Black pepper	*Filfil*
*Punica granatum* L.	Lythraceae	Pomegranate	*Jolnar*
*Qerqus infectoria*	Fagaceae	Oak apple or Oak gall	*afs*

**Table 2:** In Vitro Studies on Plants Used for Treatment of Vaginitis Mentioned in ‘‘*the Canon of Medicine*’’

**Plant**	**Part/extraction**	**Result**	**Attributable active constituent**	**References**
*Anacyclus pyrethrum* (L.)Link	Root/ Methanol (50%) extracts	Oxidative DNA damage preventive and antioxidant activity	Phenolic compounds and ascorbic acid	[9]
*Cymbopogon* *citratus*	Crude extracts	Antimicrobial effects(against acne-inducing bacteria)	-	[14]
*Iris germanica* L.	Rhizomes/methanolic extracts Aerial parts and rhizomes /ethanolic extracts	Antioxidant activity, alfa –amylase inhibition Antimicrobial(against Bacillus subtilis ATCC 6633.), antioxidant, antimutagenic activities	Isoflavonoids Phenolic compounds	[23] [24]
*Marrubium vulgare* L.	Leaves/methanol and acetone extracts Leaves/ hydroalcoholic extracts	Antioxidant activity Antioxidant and wound healing properties	Flavonoids and phenylethanoid derivatives Flavonoids and phenolic compounds	[31] [32]
*Myrtus communis* L.	Aerial parts/ aqueous and methanolic extract Leaves/myrtucommulone -containing extracts of myrtle	Antioxidant and antigenotoxic Inhibits microsomal prostaglandin PGE2 synthase-1	Myricetin-3-o-galactoside and the myricetin-3o-rhamnoside Myrtucommulone	[35] [37]
*Piper nigrum* L.	-	Suppresses Pyroptosis and Interleukin-1b Release upon ATP Triggering and Bacterial Infection/inhibited the production of E2 and NO induced by IL-1β	Piperine	[34, 45]
*Punica granatum* L.	Peels/ Ethyl acetate, acetone, MeOH, and water extracts	Radical-scavenging effect & anti-bacterial activity	Phenolic compounds	[54]
*Qerqusinfectoria Olive*.	Barks/ the MeOH, water extracts Acorns/ethyl acetate extract	High antioxidant, antiproliferative activities Moderate anti-inflammatory activities	Phenolic compounds Oleanolic triterpenes	[61] [62]

**PGE2:** Prostaglandin E2, **IL-1β:** Interlukine-1β, **NO:** Nitric oxide, **MeOH:** Methanol

**Table 3:** In Vivo Studies on Plants Used for Treatment of Vaginitis Mentioned in ‘‘*the Canon of Medicine*’’

Plant	Part used/solvent used for extraction	Method	Animal	Result	Active constituent	**References**
*Anacyclus pyrethrum* (L.)Link	Root/ Aqueous, ethanol, Chloroform extracts Root/aqueous & methanol extracts	Subplantar edema induced by carrageenan in rats/ ear edema induced by arachidonic acid in mice Acetic acid-induced writhing, hot plate, formalin tests, the mechanical allodynia were assessed in CFA-induced paw edema	Mice/rat Male mice	Anti-inflammatory activity Anti-inflammatory, antinociceptive, antioxidant effects	_ Alkaloids, Phenols	[11] [12]
*Cymbopogon* *schoenanthus* (L.) Spreng.	Essential oils	Intraperitoneal injection of casein in mice	Female mice	Suppression of neutrophil Recruitment(anti-inflammatory activity)	Citral	[18]
*Marrubium vulgare* L.	Methanolic extract	Carrageenan-induced paw edema	Male Wistar rats	Anti-inflammatory and antioxidant effects	Phenolic compounds, Flavonoids	[33]
*Myrtus communis* L.	Aerial parts/aqueous and ethanolic extracts Leaves /Ethanol extract	Hotplate , writing tests, xylene-induced ear edema, a cotton pellet tes Acetic acid-induced colonic inflammation	Mice Rat	Antinociceptive, Anti-inflammatory effects Alleviate colitis	Tannins, Alkaloids, Flavonoids Flavonoids, Phenolic compounds	[39] [40]
*Piper nigrum* L.	Ethanol extracts *-* Ethanol extracts	Ovalbumin (OVA)-induced allergic asthma model Acetic acid induced ulcerative colitis Tail immersion method, analgesia-meter, hot plate, writing tests/ carrageenan-induced paw edema	Mice Mice Rats	Ameliorated allergic inflammation Amelioration of ulcerative colitis Analgesic, Anti-inflammatory activities	- Piperine Piperine	[47] [48] [46]
*Qerqus infectoria Olive.*	Galls/ Water extract Galls /Alcoholic extract	Three different doses were administered via enema (for the acute toxicity study) Carrageenan, histamine, serotonin and prostaglandin E2 (PGE2) induced pawoedemas	Mice Male Wistar rats, male Swiss albino mice	Galls is unlikely to have significant toxicity Anti-inflammatory activity	*-* *-*	[64] [68]

**PGE2:** Prostaglandin E2

**Table 4:** Clinical Studies on Plants Used for Treatment of Vaginitis Mentioned in ‘‘*the Canon of Medicine*’’

**Plant**	**Treatment group**	**Control group**	**Study** **design**	**Number** **of patients**	**Treatment** **duration**	**Result**	**References**
*Myrtus communis* L.	Leaves vaginal gel in metronidazole base Paste containing Myrtle Fruit syrup	Vaginal gel of metronidazole Placebo paste Placebo syrup	Randomized clinical trial Double-blind, before–after RCT Double-blinded RCT	80 women 45 patients 45 patients	5 nights 6 days 3 months	Improve the efficacy of bacterial vaginosis therapy in myrtle group ↓The size of ulcers, Pain severity, The level of erythema, Exudation, Improving the quality of life in patients who suffer from RAS ↓ Mean number of vaginal bleeding days & ↓ use of pads in Myrtle group	[42] [43] [38]
*Punica granatum* L.Spreng.	Mouth rinsing with pomegranate extract(PomElla) Natural pomegranate juice Pomegranate extract Pomegranate juice	Mouth rinsing with placebo (corn muffin mix) Placebo drink Placebo drink Placebo drink	Randomized, single-blinded controlled trial Clinical trial Double-blind RCT Double-blind RCT	32 subjects (16 males, 16 females) 9 male elite weightlifters 48 participants 101 patients	4 weeks 15 days 30 days 1 year	↓Total protein and aspartate aminotransferase in the treatment group Accelerates recovery of muscle damage and soreness and inflammatory markers ↓Plasma inflammatory and oxidative stress biomarkers ↓Systemic inflammation and oxidative stress	[55] [56] [57] [58]
*Qercusinfectoria Olive.*	Topical ointment of Bensal HP (proprietary oak bark extract, Quercus rubra-3%)	SSC	Randomized, blinded controlled trial	40 patients	6 weeks	↓The Wound the diameter of the Bensal HP collective group	[69]

**RAS:** Recurrent aphthous stomatitis, **RCT:** Randomized controlled trial, **SCC:** Silver sulfadiazine cream
